# Cryoglobulins, Cryofibrinogens, and Cold Agglutinins in Cold Urticaria: Literature Review, Retrospective Patient Analysis, and Observational Study in 49 Patients

**DOI:** 10.3389/fimmu.2021.675451

**Published:** 2021-05-25

**Authors:** Katharina Ginter, Dalia Melina Ahsan, Mojca Bizjak, Karoline Krause, Marcus Maurer, Sabine Altrichter, Dorothea Terhorst-Molawi

**Affiliations:** ^1^ Department of Dermatology and Allergy, Dermatological Allergology, Allergie-Centrum-Charité, Charité - Universitätsmedizin Berlin, corporate member of Freie Universität Berlin, Humboldt-Universität zu Berlin, and Berlin Institute of Health, Berlin, Germany; ^2^ Division of Allergy, University Clinic of Respiratory and Allergic Diseases Golnik, Golnik, Slovenia; ^3^ Department of Dermatology and Venerology, Kepler University Hospital, Linz, Austria

**Keywords:** cold urticaria, cryoglobulins, cryofibrinogens, cold agglutinins, cryoproteins

## Abstract

**Introduction:**

Cryoproteins, such as cryoglobulins, cryofibrinogens and cold agglutinins, precipitate at low temperatures or agglutinate erythrocytes and dissolve again when warmed. Their pathogenetic and diagnostic importance in cold urticaria (ColdU) is unclear. In this study, we aimed to characterize the prevalence of cryoproteins in patients with ColdU.

**Methods:**

We conducted 3 analyses: i) a systematic review and meta-analysis of published data using an adapted version of the Joanna Briggs Institute’s critical appraisal tool for case series, ii) a retrospective analysis of 293 ColdU patients treated at our Urticaria Center of Reference and Excellence (UCARE) from 2014 to 2019, and iii) a prospective observational study, from July 2019 to July 2020, with 49 ColdU patients as defined by the EAACI/GA2LEN/EDF/UNEV consensus recommendations.

**Results:**

Our systematic review identified 14 relevant studies with a total of 1151 ColdU patients. The meta-analyses showed that 3.0% (19/628), 1.1% (4/357) and 0.7% (2/283) of patients had elevated levels of cryoglobulins, cryofibrinogens and cold agglutinins, respectively. Our retrospective analyses showed that cryoproteins were assessed in 4.1% (12/293) of ColdU patients. None of 9 ColdU patients had cryoglobulins, and one of 5 had cold agglutinins. In our prospective study, none of our patients had detectable cryoglobulins (0/48) or cryofibrinogens (0/48), but 4.3% (2/46) of patients had cold agglutinins (without any known underlying autoimmune or hematological disorder).

**Conclusion:**

Our investigation suggests that only very few ColdU patients exhibit cryoproteins and that the pathogenesis of ColdU is driven by other mechanisms, which remain to be identified and characterized in detail.

## Introduction

Cold urticaria (ColdU) is a subtype of chronic inducible urticaria, where wheals, angioedema or both are evoked by exposure to low temperatures (1). A reliable medical history and the results of cold stimulation tests (CSTs) such as the ice cube test or the Temp*Test* ^®^ establish the diagnosis (2).

Two different classification approaches can be found in the published literature. The classification of ColdU based on CSTs differentiates between typical ColdU, with wheals in the CSTs, and atypical ColdU, without wheals or atypical wheals in the CSTs (3). In the other approach, the etiology-based classification, a hereditary and an acquired form are distinguished, the latter being further subdivided into primary and secondary. Secondary acquired ColdU can be caused by hematologic diseases, infections, malignancies, cryoproteins (CPs) or other diseases (4). The pathophysiology of ColdU is not well understood, and autoallergic and autoimmune mechanisms, temperature-sensitive receptors, neurogenic signals, and CPs have been speculated to play a role (5, 6).

The term cryoprotein or cold protein usually includes cryoglobulins, cryofibrinogens, cold agglutinins, and sometimes other proteins. Cryoglobulins (CGs) are immunoglobulins that precipitate *in vitro* at low temperatures and dissolve when warmed (7). CGs can be divided into monoclonal (Type I) and mixed (Type II and III) immunoglobulins (8) and can cause various cold-related diseases with joint, nerve, and kidney involvement, weakness, palpable purpura, Raynaud’s phenomenon, skin rashes and ulcers (9). Various infections, especially hepatitis C, immune diseases and hematological disorders have been described as possible triggers for the occurrence of CGs (9, 10). Cryofibrinogens (CFs), which only precipitate in plasma, can occur independently or in association with CGs. Malignancies, infections and autoimmune diseases are known to induce the occurrence of CFs (11, 12). CFs can cause skin manifestations such as Raynaud’s phenomenon and purpura as well as arterial and venous thromboses (11). Cryoglobulinemia and cryofibrinogenemia, i.e. the presence of the respective CP, are distinct from cryoglobulinemic or cryofibrinogenic diseases or syndromes, i.e. resulting disease entities (11, 13). Healthy individuals may have CGs (14, 15) or CFs (11).

Cold agglutinins (CAs), also known as cold(auto)antibodies, are mostly type M immunoglobulins that agglutinate erythrocytes at low temperatures and may lead to complement activation (16).

As of now, the role and relevance of CPs in the pathogenesis of ColdU remain ill-defined and many questions need to be answered. There are several published reports on the prevalence of CPs in patients with ColdU, including many case reports and small case series. These have not been systematically analyzed at the present time, and the rates of CP-positive ColdU patients, thus, are not known. Also, it is largely unknown how often ColdU patients are assessed for CPs and what drives the decision to do so.

To answer these questions and address these unmet needs, we used a three-tiered approach. First, we performed a systematic review and meta-analysis of published reports on the rates of ColdU patients who tested positive for CGs, CFs, and CAs. Second, we retrospectively assessed how often close to 300 patients with ColdU treated at our Urticaria Center of Reference and Excellence (UCARE) (17) were assessed for these CPs and why. Finally, we prospectively measured CPs in almost 50 consecutive patients with ColdU. The overall aim of our report is to provide more clarity on the prevalence, role and relevance of CPs in ColdU.

## Materials and Methods

### Systematic Review and Meta-Analysis of Published Reports

On June 30, 2020, we conducted a systematic PubMed literature search with the terms (“cold urticaria”) AND (“cryoglobulin*” OR “cryofibrinogen*” OR “cold agglutinin*”) in accordance with the PRISMA guidelines (18) and included all published studies between 1980 and 2019 in German, English, Spanish and French. Additionally, we manually checked references in key publications on ColdU (3, 19–21), since CPs are often only reported as part of the patient description in the full text.

To be eligible, the studies had to have measured CPs in patients with ColdU diagnosed according to established criteria such as a typical patient history or positive CSTs. Accordingly, the occurrence of CPs was not allowed to be a study exclusion criterion. We excluded studies that exclusively dealt with secondary or hereditary ColdU forms and case series with fewer than 5 patients in order to avoid bias through selective reporting and publishing.

The study selection was carried out independently by two researchers based on predefined criteria. After excluding all non-relevant studies based on title and abstract screening, we procured the full texts and contacted the authors for additional information, as necessary and possible. Patient characteristics and results of CP testing were extracted independently by both researchers using a survey table. Information on inclusion criteria for the respective studies, reasons for incomplete laboratory results and reported secondary diagnoses were also collected to identify selective reporting. Where applicable, the accordance of the term “cryoglobulinemia” in the respective study with the definition used in this review was ensured. The study quality and the bias risk were then independently assessed by two researchers using a specifically adapted version of the Joanna Briggs Institute’s critical appraisal tool for case series (22) (see **Table 1**). Finally, the average percentage of CP-positive patients, i.e. patients who tested positive for CGs, CFs, or CAs, was calculated, weighted according to the respective study size.

**Table 1:** Quality assessment of the studies identified through the systematic literature review.

	1 Clear Inclusion criteria	2 Clear measurement of diagnosis	3 Clear demographics	4 If applicable: Clear reasons for not testing	5 Type of cold protein described	6 Report of secondary causes	Points(max 6)
Neittaanmäki 1985 (23)	Yes	Yes	2/3	No	Yes	Yes	4.6
Wanderer et al., 1986 (4)	Yes	Yes	2/3	All tested	Yes	Yes	5.6
Doeglas et al., 1986 (24)	Yes	Yes	2/3	All tested	Yes	No	4.6
Henquet et al., 1992 (25)	Yes	Yes	2/3	No	Yes	No	3.6
Husz et al., 1994 (26)	Yes	Yes	2/3	All tested	Yes	N/A	5.6
Koeppel et al., 1996 (27)	Yes	Yes	2/3	No	Yes	cumulative	4.6
Möller et al., 1996 (28)	Yes	Yes	2/3	No	No	No	2.6
Santaolalla Montoya et al., 2002 (29)	No	Yes	2/3	Yes (“bad conservation”)	Yes	N/A	4.6
Tosoni et al., 2003 (30)	No	Yes	2/3	All tested	Yes	Yes	4.6
Alangari et al., 2004 (31)	Yes	Yes	3/3	No	Yes	cumulative	5
Katsarou-Katsari et al., 2008 (32)	Yes	Yes	3/3	No	Yes	Yes	6
Stepaniuk et al., 2018 (33)	Yes	Yes	1/3	Not for all patients	Yes	N/A	4.3
Yee et al., 2019 (34)	Yes	Yes	2/3	No	Yes	No	3.6
Kulthanan et al., 2019 (35)	Yes	Yes	3/3	Yes (“retrospective”)	Yes	N/A	6

1. Was there clear criteria for inclusion?

2. Was the condition (ColdU) measured in a standard, reliable way for all participants included?

3. Was there clear reporting of the demographics of the participants in the study? (age, age of disease onset and ColdU duration) (1/3 point per item)

4. Did the study have all patients tested? If not: Did the study provide information about the reasons?

5. Was the outcome parameter (cold protein) clearly defined?

6. Were secondary causes of positive cryoproteins clearly reported? [N/A (not applicable) and cumulative reporting count as 1 point].

### Retrospective Analyses of ColdU Patients

We retrospectively searched the database of all chronic urticaria patients diagnosed with ColdU who presented at the UCARE at Charité-Universitätmedizin Berlin, Germany, between 2014 and 2019 for information on CPs. Collected data included patient age, laboratory workup, type of diagnosis, onset of ColdU and other diagnoses. Patient data were analyzed and reported anonymously in accordance with data protection regulations, and stored in a MS Excel Version 2019 based database.

### Prospective Assessment of ColdU Patients

Between July 2019 and July 2020, we assessed 49 patients out of 60 consecutive patients with ColdU treated at our UCARE. Of the patients originally seen during the study period (N = 60), 4 patients were excluded because the diagnosis of ColdU was not confirmed or was questionable; one patient was excluded because he no longer had active disease. In 6 patients, a sufficient amount of blood could not be obtained due to organizational reasons, patient-related reasons or laboratory difficulties. There was no control group. Our study was approved by the local ethics committee (reference EA1/069/19), and all patients provided informed consent. Inclusion criteria were 1) diagnosed ColdU as defined by the 2016 EAACI/GA^2^LEN/EDF/UNEV consensus recommendations as the “recurrence of itchy wheals and/or angioedema [ … ] reproducible in response to [ … ] cold exposure” (1), 2) disease duration of 6 weeks or longer, 3) no intake of H1 antihistamines within 3 days and/or glucocorticosteroids within 7 days prior to CSTs and blood sampling. ColdU was diagnosed in all of our patients based on their typical history and CSTs. For CSTs, the Temp*Test* 4.0^®^ and the ice cube test with 5 minutes of cold application and reading after 10 minutes were used (36). ColdU was classified as “typical” in patients who developed a wheal at the site of the CST within 10 minutes after cold exposure and as “atypical” in patients who did not (3).

We obtained and analyzed patient demographics and the course and severity of the disease including systemic reactions to cold exposure. We also assessed patients for family history for ColdU, cold-associated complaints like wheals, pruritus or angioedema and comorbidities such as atopic diseases, infections, malignancies, connective tissue disorders, thyroid diseases. Data were collected and pseudonymously entered in an MS Excel Version 2019 based database.

### Laboratory Workup

CP analyses were conducted at the central laboratory of the Charité (Labor Berlin, CGs and CFs) and the Institute of Transfusion Medicine (CAs). All peripheral-venous blood samples were collected in a standardized way with prewarmed tubes and transported directly to the laboratory, ensuring a constant transport temperature of 37°C. Since temperature deviations are a confounding factor for the analysis, a rapid and standardized transport procedure for all samples was implemented.

CAs were determined in 6ml EDTA and 10ml native venous blood, and plasma and sediment were separated at 37°C. Subsequently, samples were analyzed for CAs of blood group system I/i using foreign adult and umbilical vein erythrocytes. Reaction strength at room temperature (20°C) was evaluated by one to two observers depending on agglutination strength in categories ranging from negative (no agglutination), within physiological range (mild to moderate agglutination), borderline (more severe agglutination) to pathological/positive (massive and lumpy agglutination). At the same time, patient erythrocytes were examined with the direct Coombs test, in which in most cases only complement adhesion is expected, since any CAs detach from the erythrocytes again upon recirculation.

CFs were determined using immunoprecipitation in 2 ml EDTA, and CGs were analyzed from 2 ml serum. After centrifugation, the clear plasma or serum was examined for the formation of precipitates for 72 hours at 4°C. If precipitates or turbidity were evident, the supernatant was warmed to 37°C and, if dissolved, stored a second time at 4°C. Only in case of repeated dissolution and formation of precipitates, the sample was considered positive for CGs or CFs. Cryoprecipitates in the serum were considered as positive CGs and cryoprecipitates in the EDTA as the sum of positive CGs and positive CFs, so that both tests were always performed simultaneously for differentiation. No further differentiation or quantification of the samples was performed.

### Statistical Methods

The quantitative variables reported in this study were summarized using median, range (Min, Max) and interquartile range (IQR) using R Version 3.6.3 (37).

## Results

### Literature Review: The Studies to Date Show a Wide Scatter of Results, but on Average the Frequency of Positive CPs in ColdU Patients was Low

**Figure 1:**
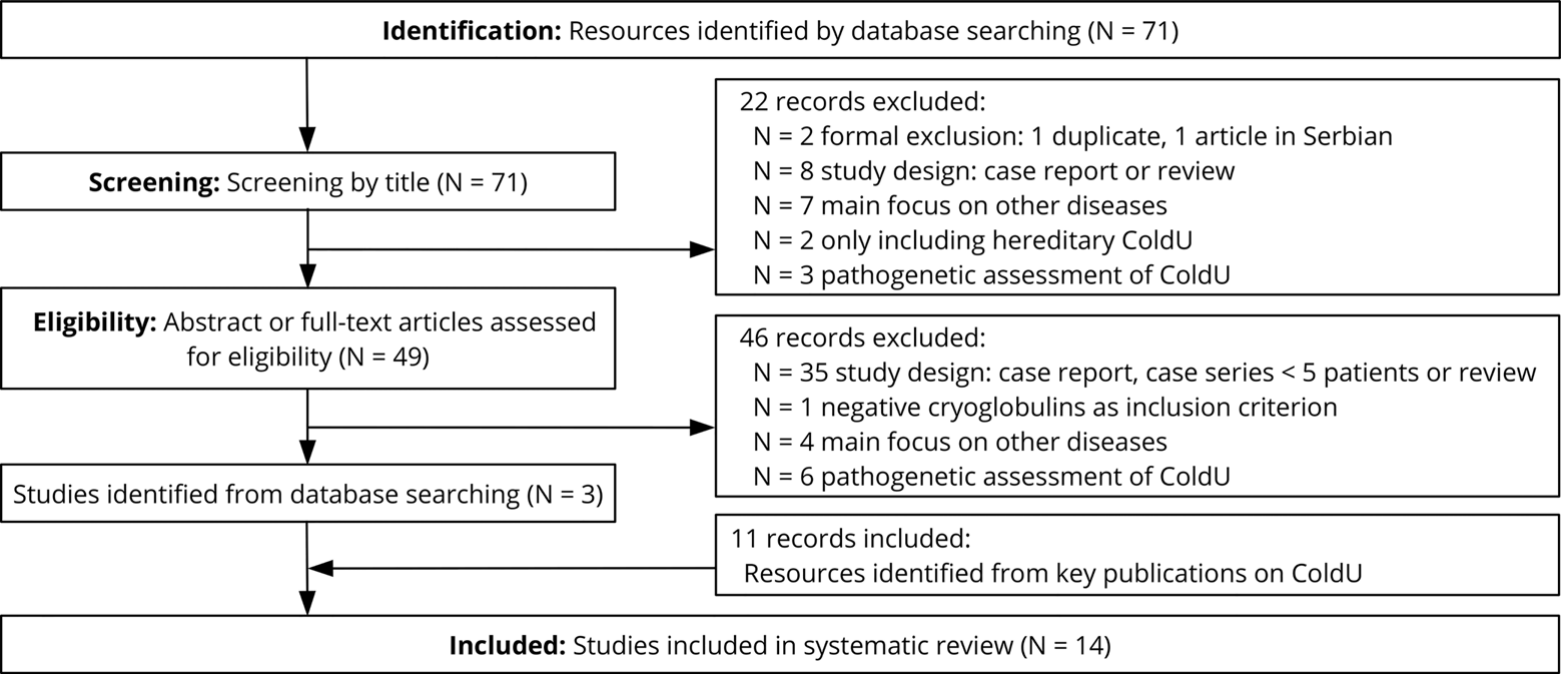
Study selection process during the systematic literature review.

Our systemic review identified 71 publications of potential relevance (**Figure 1**), of which 68 were excluded after reviewing the title, abstract or full-text. In addition, we found 11 relevant publications by checking the references of key publications, resulting in a total of 14 studies from the years 1985 to 2019 that were evaluated in this review (**Table 2**). Most reports (11 of 14) had 4 or more points out of possible 6 on the quality score and were therefore considered to be of medium or high quality (**Table 1**).

**Table 2:** Overview of the existing literature on ColdU and the rate of positive cryoproteins.

Author, Year, Country^1^, Reference	ColdU forms	N in study (M/F)	N tested: CG / CA / CF	Age^2^	Age of disease onset^2^	ColdU duration^2^	CG: %	CA: %	CF: %
Neittaanmäki, 1985, FIN (25)	All forms	220(81/139)	208 / 208 / 208	N/A	M 25.1y(R 1-74y)	M 6.3y(R 3w-37y)	2: 1.0%	0 : 0%	0: 0%
Wanderer, 1986, USA (4)	Primary, secondary, atypical	50(23/27)	50 / - / -	N/A	M 17.8y(R 3-63y)	M 4.8y(R 3m-22y)	2: 4.0%	N/T	N/T
Doeglas, 1986, NLD, (28)	No combined cold contact- and cholinergic-heat urticaria	39(14/25)	39 / 39 / -	Mdn 36y(R 10-71y)	N/A	Mdn 5y(R 4m–35y)	4: 10.3%	0: 0%	N/T
Henquet, 1992, NLD (52)	All + cold induced cholinergic urticaria	30(10/20)	18 / 14 / 5	N/A	M 26.2y(R 9-58y)	Mdn 2y(R 0-20y)	0: 0%	3 “slightly”: 21.4%	1 “slightly”: 20%
Husz, 1994, HUN (53)	Cold contact urticaria	42(14/28)	42 / 42 / -	12-63y	N/A	4m - 5y	0: 0%	0: 0%	N/T
Koeppel, 1996, FRA (34)	Superficial and deep cold urticaria	104(41/63)	72 / - / 56	N/A	M 33.5y(R 1-74y)	M 57m(R 5d-62y)	4: 5.6%	N/T	1: 1.8%
Möller, 1996, DEU (31)	All forms	56(25/31)	34 / -	M 41.0y ± SD 15.6(R 5-72y)	N/A	M 7.9y ±SD 5.8	(1: 2.9%)*		N/T
Santaolalla, 2002, ESP (30)	Probably all forms, children’s hospital	12(4/8)	9 / 7 / -	N/A	M 12.75y	M 3.5y(R 10m-8y)	0: 0%	0: 0%	N/T
Tosoni, 2003, ITA (29)	ColdU where hydroxizine and cetirizine therapy was not fitting, only positive CST	14(4/10)	14 / - / -	M 30.4y(R 11-50y)	N/A	M 48.9m(R 7-102m)	4: 28.6%	N/T	N/T
Alangari, 2004, USA (54)	Age onset <18	30 (13/17)	19 / 17 / -	Mdn 12.0y(R 2.0-19y)	Mdn 7.0y(R 0.5-14.5y)	Mdn 3.2y(R 0.5-13.5y)	0: 0%	1: 5.9%	N/T
Katsarou-Katsari, 2008, GRC (36)	No familiar ColdU	62(30/32)	50 / - / -	M 41.5y ± SD 15.6(R 20-75y)	M 32.5 ±SD 16.5(R 4-65y)	M 5.6y ±SD 3.5	2: 4%	N/T	N/T
Stepaniuk, 2018, CAN (55)	No combined urticaria	50(15/35)	16 / 15 / -	Mdn 28.5y(R 2-62y )	N/A	N/A	0: 0%	0: 0%	N/T
Yee, 2018, USA (35)	Acquired ColdU, age <19J	415(210/205)	71 / - / -	N/A	Mdn 8.0y(IQR: 4.6-12y)	N/A	1: 1.4%	N/T	N/T
Kulthanan, 2019, THA (56)	All forms, age >18J	27(6/21)	20 / 15 / 14	M 37.5y ±SD 15.0	M 34.8y ±SD 16.5	M 8.0y ± 5.6 (6 patients)	0: 0%	0: 0%	0: 0%
**Summary^3^**	**Different forms**	**1151** **(490/661)**	**628 / 357 / 283**				**19: 3.0%(R 0-28.6%)**	**4: 1.1%(R 0-21.4%)**	**2: 0.7%(R 0-20%)**

M, Male; F, Female; N, number of patients; CST, Cold Stimulation Test; d, day(s); m, month(s); y, year(s); CG, cryoglobulins; CA, cold agglutinins; CF, cryofibrinogens; CP, cryoprotein(s); N/A, information not available; N/T, Not tested; SD, Standard Deviation; R, Range; IQR, Interquartile Range; M, Mean; Mdn, Median.

^1^country code according to ISO 3166.

^2^if median and mean were given, only median was mentioned in this table.

^3^including only studies with available data, calculation: sum of positive tests in all studies divided by the total number of patients tested in all studies. *This study was not included in the summary of cryoproteins.

CPs had been determined in all 14 studies in patient cohorts ranging from 9 to 208 ColdU patients. In 0 to 28.6% (average summarized for all studies 3.0%) of the ColdU patients, positive CGs had been detected. This was reported to be associated with secondary disease in two of these patients, one of whom had lymphosarcoma (23) and one of whom had chronic lymphocytic leukemia (4). CFs had been determined in 4 studies in patient cohorts ranging from 5 to 208 ColdU patients. In 0 to 20% (summarized for all studies 0.7%) of the ColdU patients, positive CFs had been detected. CAs had been analyzed in 9 studies ranging from 7 to 208 patients. 0 to 21.4% (summarized for all studies: 1.1%) positive CAs were reported.

### Retrospective Study: In Routine Clinical Practice, Very Few ColdU Patients Were Tested for CPs, and the Rate of Positive Tests Was Low

Between 2014 and 2019, 293 patients with various forms of ColdU were seen at our UCARE. In 12 of these patients, CPs were determined. CGs were determined in 9 patients, CA were determined in 5 patients, both CG and CA were analyzed in 2 patients. CFs were not determined in any of the patients. The main reasons for CP determination were the high disease severity (in 9 of 12 patients) and suspicion of autoimmunity or hematological disease (in 3 of 12 patients). We found no anomalies in the 9 patients tested for CGs, but one of the 5 ColdU patients tested for CAs showed a positive result. However, this patient is known to have Raynaud’s syndrome, which may be associated with the presence of CAs.

### Prospective Study: Rates of CP- Positive ColdU Patients Were Very Low

Of the 49 analyzed ColdU patients, 73.5% (N = 36) were female and 26.5% (N = 13) were male, the median age was 40 years (IQR: 40 – 53 years). 69.5% (N = 34) were diagnosed with typical ColdU. Patient characteristics are described in **Table 3** and anonymized data of these patients is described in the supplementary material.

**Table 3:** Demographic characteristics of 49 ColdU patients included at Charité Berlin between July 2019 and July 2020.

		Overall (N = 49)	Negative CAs (N = 22)	Physiologic CAs (N = 22)	Positive CAs (N = 2)
**Age,** Median [Min, Max]		40.0 [14.0, 82.0]	44.5 [17.0, 67.0]	39.0 [14.0, 82.0]	23.0 [16.0, 30.0]
**Gender**	Female	36 (73.5%)	15 (68.2%)	16 (72.7%)	2 (100%)
	Male	13 (26.5%)	7 (31.8%)	6 (27.3%)	0 (0%)
**Ethnicity**	Caucasian	46 (93.9%)	22 (100%)	21 (95.5%)	2 (100%)
	Latino/Hispanic	1 (2.0%)	0 (0%)	1 (4.5%)	0 (0%)
	Middle East	2 (4.1%)	0 (0%)	0 (0%)	0 (0%)
**Age of onset of the disease,** Median [Min, Max]		31.0 [0, 81.0]	28.0 [0, 64.0]	34.5 [10.0, 81.0]	21.0 [12.0, 30.0]
**Time since onset of symptoms in months,** Median [Min,Max]		60.0 [3.00, 600]	95.0 [4.00, 600]	37.0 [3.00, 480]	26.0 [4.00, 48.0]
**Cold-induced reactions ≤ 12 months**					
Pruritus		49 (100%)	22 (100%)	22 (100%)	2 (100%)
Wheals		40 (81.6%)	18 (81.8%)	18 (81.8%)	2 (100%)
Angioedema		28 (57.1%)	12 (54.5%)	13 (59.1%)	2 (100%)
**Cold stimulation tests**	Ice Cube Test positive	31 (63.3%)	14 (63.6%)	15 (68.2%)	0 (0%)
	Temp*Test* positive	33 (67.3%)	15 (68.2%)	15 (68.2%)	1 (50.0%)
**Cryoproteins**					
**Cryoglobulins**	Negative	48 (98.0%)	–	–	–
	Not determined*	1 (2.0%)	–	–	–
**Cold agglutinins**	Positive	2 (4.1%)	–	–	–
	Negative	22 (44.9%)	–	–	–
	Within physiological range	22 (44.9%)	–	–	–
	Not determined*	3 (6.1%)	–	–	–
**Cryofibrinogens**	Negative	48 (98.0%)	–	–	–
	Not determined*	1 (2.0%)	–	–	–
**Diagnosis**					
Typical cold urticaria		34 (69.4%)	16 (72.7%)	16 (72.7%)	1 (50.0%)
Atypical cold urticaria		3 (6.1%)	2 (9.1%)	0 (0%)	1 (50.0%)
Probably atypical cold urticaria		10 (20.4%)	3 (13.6%)	6 (27.3%)	0 (0%)
Cholinergic cold urticaria		1 (2.0%)	1 (4.5%)	0 (0%)	0 (0%)
Cold-induced pruritus		1 (2.0%)	0 (0%)	0 (0%)	0 (0%)
Cold-induced anaphylaxis		23 (46.9%)	11 (50.0%)	10 (45.5%)	1 (50.0%)
First-degree relatives with ColdU		3 (6.1%)	1 (4.5%)	1 (4.5%)	0 (0%)
**Past medical History**					
Raynaud’s syndrome		7 (14.3%)	3 (13.6%)	2 (9.1%)	0 (0%)
Lip cyanosis after cold exposure		7 (14.3%)	5 (22.7%)	0 (0%)	0 (0%)
Asthma bronchiale		6 (12.2%)	3 (13.6%)	3 (13.6%)	0 (0%)
Allergic rhinitis/conjunctivitis, atopic dermatitis		20 (40.8%)	10 (45.5%)	8 (36.4%)	0 (0%)
Current or previous malignancies		1 (2.0%)	0 (0%)	1 (4.5%)	0 (0%)
Thyroid disorders		12 (24.5%)	5 (22.7%)	5 (22.7%)	1 (50.0%)
Connective tissue disorders		2 (4.1%)	1 (4.5%)	1 (4.5%)	0 (0%)
Systemic reaction after hymenoptera sting		7 (14.3%)	3 (13.6%)	2 (9.1%)	1 (50.0%)

*Sample not suited for analysis.

N, number of patients; CAs, Cold agglutinins.

None of 48 patients tested positive for CGs or CFs, and 2 of 46 patients (4.3%) tested positive for CAs. The two patients with positive CAs were female and aged 30 and 16 years, respectively. Both reported having experienced pruritus, wheals and angioedema within the 12 months prior to study inclusion. One woman had been diagnosed with classic acquired (ice cube test negative, Temp*Test* positive) and the other with atypical ColdU (IceCubeTest and Temp*Test* negative). The classic acquired ColdU patient also reported cold-induced anaphylaxis and breathing difficulties in response to cold exposure, suffered from hypothyroidism and has experienced a systemic reaction after a hymenoptera sting. The atypical ColdU patient reported no further diseases or systemic reactions. Information on the clinical differences between patients with physiological, elevated, and undetectable CAs is shown in **Table 3**. We could not detect major differences between patients with physiological and undetectable CAs.

## Discussion

In this study, we showed that the frequency of positive CPs in ColdU patients was low with all three methods used. Moreover, ColdU patients were rarely assessed for CPs in routine clinical practice and testing positive was rarely linked to clinical features or consequences. Our results discourage routine clinical testing of ColdU patients for CPs and call for further characterization of the role and relevance of CPs in ColdU patients who test positive.

### Comparison with Previous Literature

When compared to previous publications, the findings of our literature review are consistent with the review by Alain Claudy, with whose dataset we calculated a proportion of 2% positive CGs in ColdU patients (20). Contradictory results with a proportion of 20% positive CGs in ColdU patients were reported in the review by Houser et al. (38), which was cited in the teaching book by Czarnetzki (39). In order to summarize all publications until 1970, Houser et al. divided the number of publications with ColdU patients and cryoglobulinemia by the total number of publications with ColdU patients. Since the majority of the included studies are case reports, their result can be explained by the distortion due to a presumably high reporting and publication bias.

The frequency of positive CGs in the studies identified through our systematic literature review ranged between 0% and 28.6%, with strikingly high values in Doeglas (24), 10.3%, and Tonsoni (30), 28.6%. The high percentage in Doeglas et al. can be plausibly explained by the different study design, as it is the only study with a measurement of CPs in patients at 3-6 different points in time. The high proportion in Tosoni et al. of 4 patients with slightly positive CGs (28.6%) can only be partly attributed to the small number of cases (N = 14) and remains largely unexplained.

### Limitations

When evaluating the literature on CPs and ColdU, limitations on several levels must be considered: On the one hand, distortion by a reporting bias should be considered, meaning that purely negative laboratory tests and cases with negative CGs may not be reported or published. This effect is reflected in the fact that the proportion of positive CGs in the case reports is much higher and decreases with increasing sample size.

The heterogeneity of the studies included in this review is a further potential constraint when summarizing the literature (see **Table 1**). Two studies (29, 30) did not clearly report their inclusion criteria and one (28) did not differentiate between the different CPs (28). Moreover, 7 studies did not explain the reasons for their incomplete laboratory results. Different authors included and excluded different forms of ColdU, while others did not define criteria at all. None of the papers reported on the handling of blood samples or on the laboratory procedure used, which could lead to distortions due to different measurement standards (7) and reference values (40) in different countries, as a survey of 137 European laboratories showed (41). Moreover, most publications did not report on possible underlying diseases in CP-positive patients, so that we could not distinguish between primary and secondary cryoproteinemia.

The validity of the prospective and retrospective study might be limited by the specific patient selection at our UCARE: Patients who had already been seen by a specialist before study enrollment may have been given a different diagnosis or referred directly to other departments, such as hematology, as a result. Additionally, because this study was conducted at a tertiary hospital, patients with particularly severe ColdU may have been primarily enrolled in the study. In addition, due to the study design, we could not reconstruct the exact blood collection and transport logistics in the retrospective analysis.

Nonetheless, this study analyzed a substantial number of well-characterized ColdU patients with quality-controlled sampling and transport, and only 5 other studies known to us had included similar or higher numbers of patients (4, 23, 27, 32, 34).

### Explanatory Approaches for the Low Frequency of CPs and the Large Scatter of Results of Previous Publications

Our results raise the question of why, on the one hand, such disparate results were reported in the literature and why, on the other hand, we observed such a low frequency of CPs.

First, according to a theory by Wanderer (42), many laboratory results on CP could be false negatives and the actual true frequency of positive CP in ColdU patients might be much higher. However, this theory is contradicted by the fact that all studies known to us involving a large number of patients reported a very low percentage of positive CP [such as (23, 27, 34)].

More likely, therefore, is the theory that there are two manifestations of ColdU: a CP-negative and a much rarer CP-positive form. This theory is supported by the numerous case reports of patients suffering from CG-positive ColdU and additionally other diseases such as infections (38, 43–50) or hematologic disorders/malignancies (5, 51–55). In these patients, the underlying disease could have caused cryoproteinemia and as a consequence ColdU. It is possible that the underlying disease may also be diagnosed later, as Polliack and Lugassy found out (56). Furthermore, it is conceivable that the symptoms and the clinical course of CP-positive and CP-negative ColdU differ, although to our knowledge there have been no publications on this subject to date.

Another explanatory approach is that the presence of CP in a patient and the occurrence of ColdU could be completely independent of each other. To verify this theory, a comparison with the frequency of positive CPs in the general population would be helpful, but we could not find any published data on this. Furthermore, it seems possible that a third disease has caused both cryoglobulinemia and ColdU in CP-positive patients. In this case, CPs might have a diagnostical, but only an indirect pathogenical relation to ColdU.

### Implications

Overall, we have found that CPs are rare in ColdU patients, suggesting that physicians may limit the measurement of CPs even more to patients with a clinical suspicion of secondary ColdU. In addition to cost savings, this automatically leads to fewer false positive findings and fewer unnecessary blood samples taken from patients. It remains to be clarified to what extent positive CPs have a diagnostic and therapeutic benefit for ColdU patients or to what extent they influence the course of the disease or the patients’ symptoms. Furthermore, we recommend the development of an international standardized protocol and defined threshold values for CP detection to reduce false negative and false positive results. The pathomechanism of ColdU with and without CPs continues to remain unclear and requires further research.

### Conclusion

In summary, both through our literature review and through the analysis of our patient data, we could show that only few ColdU patients exhibit CPs and that the pathogenesis of ColdU might mainly be driven by other mechanisms, which remain to be identified and characterized in detail.

## Data Availability Statement

The raw data was generated at Charité — Universitätsmedizin Berlin, Germany. All data shown in the study is in the **Supplementary Material**, further data supporting the findings of this study is available from the corresponding author on request.

## Ethics Statement

Ethical approval and consent details: Ethical approval was obtained from the ethics committee of the Charité – Universitätsmedizin Berlin, number EA1/069/19. The patients/participants provided their written informed consent to participate in this study.

## Author Contributions

KG performed the statistical analysis and drafted the manuscript. DA, KK, and SA were involved in patient recruitment and proof-reading of the manuscript. MM and MB were involved in study planning and proof-reading of the manuscript. DT-M has planned the study, coordinated the study, collected patient data, was involved in statistical analysis and drafted the manuscript. All authors contributed to the article and approved the submitted version.

## Funding

Open access publication fund of Charité Universitätsmedizin Berlin.

## Conflict of Interest

KK is or recently was a speaker and/or advisor for and/or has received research funding from Berlin Chemie, CSL Behring, Moxie, Novartis, Roche and Shire/Takeda. SA is or recently was a speaker and/or advisor for and/or has received research funding from AstraZeneca, Allakos, Sanofi, Moxie and Novartis. MM is or recently was a speaker and/or advisor for and/or has received research funding from Allakos, Amgen, Aralez, ArgenX, AstraZeneca, Celldex, Centogene, CSL Behring, FAES, Genentech, GIInnovation, Gilead, Innate Pharma, Kyowa Kirin, Leo Pharma, Lilly, Menarini, Moxie, Novartis, Roche, Sanofi/Regeneron, Third HarmonicBio, UCB, and Uriach. MB is or recently was a speaker and/or advisor for Novartis.

The remaining authors declare that the research was conducted in the absence of any commercial or financial relationships that could be construed as a potential conflict of interest.

## Abbreviations

CA(s), Cold agglutinin(s); CF(s), Cryofibrinogens; CG(s), Cryoglobulin(s); ColdU, Cold Urticaria; CP(s), Cryoprotein(s); CST(s), Cold stimulation test(s); UCARE, Urticaria Center of Reference and Excellence.
