# Supplementary material for: Cryoglobulins, Cryofibrinogens, and Cold Agglutinins in Cold Urticaria: Literature Review, Retrospective Patient Analysis, and Observational Study in 49 Patients
Source: Front Immunol. 2021 May 25;12:675451. doi: 10.3389/fimmu.2021.675451 (PMC8186313; doi:10.3389/fimmu.2021.675451)
Supplement: Supplementary file 1 [file DataSheet_1.pdf]

Supplementary Material: Complete table of anonymized patient data of 60 ColdU patients at Charité Berlin between July 2019 and July 2020

ID	excluded	reasons for exclusion	reasons for incomplete laboratory results	CG	CA	CF	IceCubeTest	TempTest	Diagnosis	Cold-induced Anaphylaxis	First-degree relatives with Cold Urticaria	Age	Gender	Ethnicity	Age of onset of the disease	Time since onset of symptoms in months	Cold-induced pruritus in last 12 month	Cold-induced wheals in last 12 month	Cold-induced angioedema in last 12 month	Other cold-induced reaction in last 12 month	Raynaud's syndrome	Lip cyanosis after cold exposure	Asthma bronchiale	Allergic Rhinitis/conjunctivitis, Atopic dermatitis	Current or Previous Malignancies	Thyroid disorders	Connective tissue disorders
**1**	-			-	phys	-	+	+	CC	1	0	41	F	3	39	26	1	1	1	Yes	0	0	0	1	0	0	0
**2**	-		L	N/T	-	N/T	+	+	CC	0	0	26	M	3	23	10	1	0	0	Yes	0	0	0	1	0	0	0
**3**	-			-	phys	-	+	+	CC	0	0	39	F	3	36	38	1	1	0	Yes	1	0	0	0	0	0	0
**4**	-			-	-	-	+	+	CC	0	0	56	F	3	46	123	1	1	1	Yes	0	0	0	1	0	0	0
**5**	-			-	phys	-	+	+	CC	0	0	77	M	3	71	72	1	1	0	No	0	0	0	0	0	0	0
**6**	-			-	-	-	-	-	AC	0	0	27	M	3	22	60	1	1	0	No	0	0	0	1	0	0	0
**7**	-			-	phys	-	+	+	CC	1	0	56	F	3	20	432	1	1	1	Yes	0	0	0	0	0	1	1
**8**	-			-	-	-	+	+	CC	0	0	42	F	3	42	4	1	1	0	Yes	0	1	1	1	0	1	0
**9**	-			-	-	-	+	+	CC	1	0	48	F	3	0	576	1	1	1	Yes	0	0	0	1	0	1	0
**10**	-			-	phys	-	+	-	CC	0	0	32	M	3	32	9	1	1	1	Yes	0	0	1	1	0	0	0
**11**	-			-	phys	-	+	+	CC	0	0	36	F	3	31	60	1	1	0	No	0	0	0	1	0	0	0
**12**	-			-	phys	-	+	+	CC	1	0	30	M	3	28	27	1	1	1	Yes	0	0	0	1	0	0	0
**13**	-			-	phys	-	+	+	CC	1	0	25	M	3	24	9	1	1	1	Yes	0	0	0	0	0	0	0
**14**	-			-	phys	-	-	+	CC	0	0	64	F	3	64	3	1	0	0	No	0	0	0	1	1	1	0
**15**	-			-	-	-	+	+	CC	1	0	67	F	3	64	36	1	1	1	Yes	0	0	0	0	0	1	0
**16**	-			-	-	-	+	+	CC	1	0	53	M	3	47	144	1	1	1	No	0	1	0	0	0	0	0
**17**	-			-	phys	-	+	+	CC	0	0	82	F	3	81	14	1	0	0	No	0	0	0	0	0	1	0
**18**	-			-	-	-	+	+	CC	1	0	66	F	3	64	24	1	0	1	Yes	1	0	0	1	0	0	0
**19**	-			-	-	-	-	-	AC	0	0	36	F	3	11	600	1	1	1	Yes	1	0	0	0	0	0	0

**20**	**-**			-	-	-	-	+	CC	1	0	66	F	3	60	147	1	0	0	No	0	0	0	0	0	1	0
**21**	**-**			-	-	-	-	+	CC	0	0	53	F	3	1	52	1	0	0	No	0	0	1	1	0	0	0
**22**	**-**			-	-	-	-	-	pAC	0	0	45	F	3	38	84	1	1	1	No	0	0	0	1	0	0	0
**23**	**-**			-	-	-	+	+	CC	0	1	17	M	3	11	76	1	1	1	No	0	0	0	0	0	0	0
**24**	**-**			-	phys	-	+	+	CC	0	0	55	F	3	54	14	1	1	1	Yes	0	0	0	0	0	1	0
**25**	**-**			-	-	-	+	+	CC	0	0	29	F	3	16	180	1	1	1	No	0	0	0	0	0	0	0
**26**	**-**			-	phys	-	-	-	pAC	0	0	38	F	3	28	120	1	1	1	Yes	0	0	0	0	0	0	0
**27**	**-**			-	-	-	+	-	ChC	1	0	28	F	3	14	168	1	1	0	No	0	0	1	1	0	0	1
**28**	**-**			-	-	-	-	-	pAC	1	0	66	M	3	41	300	1	1	0	No	1	0	0	0	0	0	0
**29**	**-**			-	phys	-	+	+	CC	1	0	40	M	3	37	49	1	1	1	Yes	0	0	0	0	0	0	0
**30**	**-**			-	-	-	+	+	CC	1	0	60	F	3	30	360	1	1	1	No	0	0	0	0	0	0	0
**31**	**-**		B	-	N/T	-	-	-	pAC	0	0	31	F	1	25	72	1	1	0	No	1	1	0	1	0	0	0
**32**	**-**			-	-	-	+	+	CC	1	0	29	M	3	26	41	1	1	1	Yes	0	1	0	0	0	0	0
**33**	**-**			-	-	-	+	-	CC	0	0	43	M	3	25	300	1	1	0	Yes	0	0	0	0	0	0	0
**34**	**-**			-	phys	-	-	-	pAC	1	0	19	F	3	10	108	1	1	0	Yes	1	0	0	0	0	0	0
**35**	**-**			-	phys	-	-	-	pAC	1	0	52	F	3	34	216	1	0	1	Yes	0	0	0	0	0	0	0
**36**	**-**			-	+	-	-	+	CC	1	0	30	F	3	30	4	1	1	1	Yes	0	0	0	0	0	1	0
**37**	**-**			-	phys	-	+	+	CC	0	0	14	F	3	10	48	1	1	0	No	0	0	1	1	0	0	0
**38**	**-**			-	-	-	-	+	CC	0	0	44	F	3	36	96	1	1	0	No	0	1	0	0	0	0	0
**39**	**-**		B	-	N/T	-	+	+	CC	1	1	2	F	1	21	28	1	0	1	Yes	0	0	0	1	0	0	0
**40**	**-**			-	phys	-	+	+	CC	0	0	30	F	2	24	72	1	1	0	No	0	0	1	1	0	0	0
**41**	**-**			-	+	-	-	-	AC	0	0	16	F	3	12	48	1	1	1	Yes	0	0	0	0	0	0	0
**42**	**-**			-	phys	-	-	-	pAC	1	0	45	F	3	42	36	1	0	1	Yes	0	0	0	0	0	0	0
**43**	**-**			-	phys	-	+	+	CC	1	0	25	F	3	25	11	1	1	1	Yes	0	0	0	1	0	0	0
**44**	**-**		L	-	N/T	-	+	+	CP	0	0	52	F	3	50	24	1	1	0	Yes	1	1	0	0	0	1	0
**45**	**-**			-	-	-	+	+	CC	1	0	30	F	3	24	72	1	1	1	Yes	0	1	0	0	0	0	0
**46**	**-**			-	phys	-	+	+	CC	1	0	39	M	3	39	6	1	1	1	Yes	0	0	0	0	0	1	0
**47**	**-**			-	-	-	-	-	pAC	1	0	50	F	3	42	94	1	1	0	No	0	0	0	1	0	1	0
**48**	**-**			-	phys	-	-	-	pAC	0	1	36	F	3	35	16	1	1	1	No	0	0	0	0	0	0	0
**49**	**-**			-	phys	-	-	-	pAC	0	0	56	F	3	36	480	1	1	0	No	0	0	0	0	0	0	0
**50**	**1**	nl	O	N/T	N/T	N/T	+	+	CC	1	0	39	M	3	29	240	1	1	0	Yes	0	0	0	0	0	0	0

**51**	**1**	nl	L	N/T	N/T	N/T	+	+	CC	1	0	51	F	3	49	24	1	1	0	Yes	0	0	1	0	0	0	0
**52**	**1**	na		-	-	-	-	-	N/A	0	0	27	M	3	36	9	1	1	0	No	0	0	0	1	0	0	0
**53**	**1**	d		-	-	-	N/A	N/A	N/A	N/A	N/A	N/A	N/A	N/A	N/A	N/A	N/A	N/A	N/A	N/A	N/A	N/A	N/A	N/A	N/A	N/A	N/A
**54**	**1**	nl	B	N/T	N/T	N/T	-	-	pAC	0	0	9	M	3	8	2	1	1	1	No	0	0	0	0	0	0	0
**55**	**1**	d		-	phys	-	-	-	N/A	0	0	25	F	3	15	120	0	0	1	Yes	0	0	0	1	0	0	0
**56**	**1**	nl	O	N/T	N/T	N/T	-	-	pAC	1	0	39	F	3	11	336	1	0	1	Yes	0	1	0	0	0	0	0
**57**	**1**	nl	B	N/T	N/T	N/T	-	-	AC	N/A	1	18	F	3	16	23	1	1	1	Yes	0	1	1	0	0	0	0
**58**	**1**	d	B	N/T	-	-	N/A	N/A	N/A	N/A	N/A	56	M	3	46	120	1	1	1	Yes	N/A	N/A	N/A	N/A	N/A	N/A	N/A
**59**	**1**	d	B	-	N/T	-	-	-	N/A	0	1	17	F	1	3	168	1	1	1	No	0	1	0	1	0	0	0
**60**	**1**	nl	O	N/T	N/T	N/T	-	-	pAC	1	1	14	F	3	6	96	1	0	1	Yes	0	1	0	1	0	0	0

Legend:
Laboratory results: CF Cryofibrinogens, CG Cryoglobulins, CA Cold agglutinins, phys = within physiological range
resasons for exclusion: nl = no laboratory results, na = no active disease, d = diagnosis other than cold urticaria
reasons for incomplete lab results: O organizational, L Laboratory error, B insufficient blood amount
Diagnosis: CC = Classical acquired ColdU, AC = Atypical ColdU, pAC = probably Atypical ColdU, ChC = Cholinergic ColdU, CP = Cold-induced Pruritus, HC = Hereditary ColdU
Ethnicity: 1 Middle_East, 2 Latino/Hispanian, 3 Caucasian
N/A = information not available
